# Prevalence of occupational injuries among workers in the iron and steel industries in Tanzania

**DOI:** 10.5334/aogh.4503

**Published:** 2025-02-06

**Authors:** Saumu Shabani, Bente Elisabeth Moen, Wakgari Deressa, Simon Henry Mamuya

**Affiliations:** 1 Department of Environmental and Occupational Health, School of Public Health and Social Sciences, Muhimbili University of Health and Allied Sciences, Tanzania; 2 University of Bergen, Norway; 3 Department of Preventive Medicine, School of Public Health, College of Health Sciences, Addis Ababa University, Ethiopia

**Keywords:** Occupational injuries, Iron and steel, Industries

## Abstract

*Background:* In Tanzania, iron and steel manufacturing industries are based on manual work with minor automation, as workers segregate scrap metals and add them to furnaces for melting. The workers here are exposed to hazardous conditions, posing a risk to their health.

*Objective:* To determine the prevalence of occupational injuries and possible predictors for injuries among workers in the iron and steel industries in Tanzania.

*Methods:* The cross‑sectional study was conducted in 2022 in Tanzania. Workers from the production lines in four iron and steel factories participated in the study. The data were collected by interviews, using a structured questionnaire modified from the International Labour Organization (ILO) manual on occupational injury statistics from household surveys and establishment surveys. Chi‑squared tests and regression analyses were used.

*Results:* Out of 381 invited workers, 321 participated in the study (response rate: 84). Of the respondents, 209 had experienced at least one injury that restricted them from work at least one day in the past year, giving an overall prevalence of occupational injuries of 65.1% per year. Out of the injured respondents, 135 (64.6%) reported being hospitalized or lying on the bed at home due to the injury. Working years, working hours per day, working 12‑hour shifts, and their section at the workplace (rolling mill or furnace) were factors significantly associated with occupational injuries in univariate regression analyses. Working hours of more than 10 hours per day, adjusted for all other factors, gave an odds ratio of 2.54 for experiencing injuries at work, with a 95% confidence interval (1.46–4.41), while no other factors showed significant association with injuries after adjustment.

*Conclusion:* The prevalence of occupational injuries in the Tanzanian iron and steel industries was 65.1%. Working for more than 10 hours per day was a significant predictor of occupational injuries.

## Introduction

The iron and steel industries are crucial for the development of any country. Iron and steel production provides essential raw materials for many other industries. These materials are among the most widely used globally, with an annual production of 1.6 billion metric tons and the most recycled material in the world. This makes significant contributions to the gross domestic product (GDP) of producing countries [1]. The work environment in the iron and steel industries can pose several risks of injuries to workers due to the use of mechanical equipment and energy‑intensive processes. Iron and steel production involves many potential hazards for occupational injuries, such as high temperatures, working at heights, working in confined spaces, the use of moving and unguarded machinery, the presence of sharp metal parts, and heavy lifting [2]. These risk factors can lead to occupational accidents.

According to estimates from the International Labour Organization (ILO), 380,500 workers die from occupational accidents every year, and 374 million workers suffer from non‑fatal occupational accidents [3, 4]. It is estimated that lost workdays globally due to occupational accidents represent almost 4% of the world’s GDP, and in some countries, the estimate rises to 6% or more [4]. However, in many countries, the numbers of workplace accidents, fatalities, and diseases are not reported or recorded [5]. Therefore, global figures can only be estimated. Most work‑related deaths and non‑fatal occupational accidents may occur in low‑ and middle‑income countries [4]. Occupational accidents not only cause enormous pain, suffering, and death among the injured workers but also affect their families, workplaces, and society. Occupational accidents result in various consequences, such as loss of skilled and unskilled workers, material loss (including damage to machinery and equipment), medical care costs, and compensation payments [6].

Occupational injuries are among the most common health problems in the iron and steel industries. Workers in these industries are at greater risk for both fatal and non‑fatal injuries compared with those in other manufacturing sectors. Globally, occupational injuries in the iron and steel industries are reported to be significantly higher than in other manufacturing industries [2, 7–15]. This is likely due to the complex nature of production processes and material handling involved in iron‑ and steelmaking [16].

Recently, the Tanzanian government announced a focus on increasing investments in manufacturing industries to drive economic growth. This initiative is expected to boost the development of the iron and steel industries. Most of Tanzania’s iron and steel manufacturing processes are manual, requiring workers to directly engage in tasks such as collecting, sorting, and adding scrap metal, and melting it in furnaces. However, there is a significant lack of personal protective equipment (PPE) such as helmets, clothing, safety shoes, and gloves. Most workers in Tanzania’s iron and steel sectors, particularly those on production lines, have low socioeconomic status and low skill levels. They are typically young males with weak communication skills and limited on‑the‑job training experience. These factors increase their risk of injury [17].

To our knowledge, no study has been conducted on occupational injuries in Tanzania’s iron and steel industries. Only a few studies have been done on occupational injuries and fatalities in Tanzania, including one in tanzanite mines [18], one among solid waste collectors [19], and one reporting work‑related injuries registered in the workers’ compensation funds [20]. Due to this lack of information, there is a need for data on occupational injuries.

This study aims to gather information directly from the workers to understand the safety conditions within the workplace and to determine whether improvements or mitigations are needed. The study seeks to determine the prevalence of occupational injuries and identify possible predictors of occupational injuries among workers in the iron and steel industries in Tanzania.

## Material and Methods

### Study setting and design

This cross‑sectional study was conducted from July 2022 to September 2023 in Dar es Salaam and the coastal region of Tanzania. These two regions were selected owing to the availability of a large number of factories in the iron and steel industries here, compared with the other regions in Tanzania. Four factories were randomly selected for participation from a list of factories in the iron and steel industries, provided by the Occupational Safety and Health Authority in Tanzania. Two factories were in Dar es Salaam and two in the coastal region.

### The iron and steel manufacturing process

The manufacturing process in all four participating iron and steel factories is divided into two main, separate sections: a furnace section and a rolling mill section.

- In the furnace section, metal scrap brought to the industry site is melted in the induction furnace to form molten steel. The floating furnace slag is removed by raking. The molten steel is then poured into a large ladle and subsequently into smaller crucibles and molds of varying sizes to form steel billets. These billets are cooled and manually weighed before being processed in the rolling mill. This section presents several potential risk factors for injuries, including machinery, gas exposure, hot substances, and the sharp edges of scrap metals. The workers are involved in sorting scrap metal, manually handling steel billets, and feeding metal scraps into the furnace oven. All these activities expose workers to accidents and injuries, such as cuts and burns.
- The rolling mill process involves heating steel billets to approximately 960 °C in a gas furnace. The billets are then transferred as red‑hot bars to a roughing machine, where they are shaped and lengthened to thin bars. Electric motor‑operated conveyor rails transport the hot metal billets between machines. The red‑hot bars are manually fed into arranged rolling mill machines. The final metal bars are then moved into a cooling bed and cut into different sizes. Potential risk factors for injuries in this section include heavy metal parts, hot substances, and moving machinery parts. Workers in the rolling section may experience burns while transferring the billets to the conveyor line or when attempting to cross roller conveyors. Additionally, workers can be injured by hitting the steel bar if it fails to move on the conveyor rail.

### Sample size

The sample size was determined by calculating the size of the population needed to estimate the prevalence of injuries in this working population, using the program Open Epi. The population size used (for the finite population correction factor [fpc]) was 1000. The hypothesized percentage (%) frequency of outcome factor in the population was 33% according to the study of work‑related injuries and associated risk factors among iron and steel industry workers in Addis Ababa, Ethiopia [2]. The sample size confidence interval (CI) was 95%. The calculated sample size was *n* = 381, with a significance level of 0.05 and a statistical power of 85.

### Selection of participants

Participants were workers in the production line in the four factories, and a total sample of 381 were invited, as suggested by the sample size calculation. The total sample of 381 was proportionally obtained from the number of workers in the production line of each of the four factories. Industry A had 125 workers in the production line, and we randomly selected 99 participants from this group. Similarly, industry B had 76 production workers, and 60 workers were selected; industry C had 116 production workers, and 92 were selected; and industry D had 165 workers, and 130 were selected. The response rate was 84 in total; it was 100% in industries A, B, and C, while industry D had a response rate of 54 (Table 1).

**Table 1:** Characteristics of study participants (*n* = 321).

VARIABLE	COUNT (NUMBER)	PERCENTAGE (%)
***Industry***		
A	99	31
B	60	19
C	92	29
D	70	21
***Section***		
Furnace	139	43.3
Rolling mills	182	56.7
***Age (years)***		
18–30	179	56
31–44	107	33
≥ 45	35	11
***Education***		
Never been in school	19	5.9
Primary school	133	41.4
Secondary school and above	169	52.6
***Marital status***		
Never married	83	25.9
Married	238	74.1
***Working years***		
1–4 years	245	76.3
> 4 years	76	23.7
***Working hours per day***		
≤ 10 hours	77	24
> 10 hours	244	76
***Shiftwork***		
Permanent night shift or rotating shift schedule	184	57.3
Only daytime work	137	42.7

### Data collection tool, variables, and procedures

A structured questionnaire modified from an ILO manual on methods for occupational injury statistics from household surveys and establishment surveys [21] was used to obtain information on occupational injuries and personal and work factors that might be associated with a risk of injuries. The questionnaire was translated from English to the Swahili language and back again. Before the actual data collection, a pre‑test was conducted with 5% of the total sample size in another factory not participating in the present study, and a few corrections were made afterward. A few questions needed clarification, with more words for explanations. A research assistant with a bachelor’s degree in sociology, who had experience with data collection, was trained for 3 days to collect the data by interviewing each worker. The interviews took place at the worksite in a private room, and an interview lasted 20–30 minutes. The data collector reported and discussed the data collected daily with the principal investigator throughout the whole data collection period, and made sure that all questions were answered.

The main outcome variable was the presence of occupational injuries among iron and steel industry workers over the past year. This was specifically defined as all accidents that had caused injury or disease at the workplace or elsewhere while the worker was performing tasks for the employer [21]. Additionally, the injury must have incapacitated the worker for least 1 day, excluding the day of the accident [21].

Other independent variables asked for were socio‑demographic; age, sex, marital status, educational level, and information about the work; number of working years; work hours per day; work section (furnace or rolling mill); and status as a shift worker (yes/no). Shiftwork in this study means working on a permanent night shift or on a rotating shiftwork schedule, including both night and afternoon (Table 1). The night shift was defined as work at night (between midnight and 5 a.m.) in an industry. In addition, the workers were asked for the number of occupational injuries experienced, the time when the injury occurred, hospitalization, the location at which the injury happened, the body part affected, and the type of injury they had experienced (Table 2).

**Table 2:** Description of occupational injuries among 321 workers from iron and steel industries in the preceding 12 months.

OCCUPATIONAL INJURY DESCRIPTION	COUNT	PERCENTAGE (%)
*Injury prevalence rate for the preceding 12 months*	221	68.8
*Occupational injury (restricted from work at least 1 day)*	209	65.1
***Frequency of injuries in the preceding 12 months (among the injured people [n = 209]***		
Once	89	42.6
Twice	52	24.9
Three times	39	18.7
Four times/more	29	14.3
***Time when the injury occurred***		
Morning	20	9.6
Afternoon	121	57.9
Evening	33	15.8
Midnight	23	11.0
I don’t remember	12	5.7
*Workers hospitalized*	135	64.6
***Number of days hospitalized***		
1–6 days	103	49.3
7–14	22	10.5
15–21	7	3.3
≥ 30 days	3	1.4
***Location where the accident occurred***		
At usual work area in the factory	192	91.9
Somewhere else in the factory/unit	15	7.2
On work‑related travel	2	0.9
***Parts of the body affected***		
Upper extremities	88	42.1
Lower extremities	58	27.8
Whole body/multiple sites equally injured	26	12.4
Head	16	7.7
Back	15	7.2
Neck	4	1.9
Trunk/internal organs	2	0.9
***Type of injury***		
Superficial injuries	110	52.6
Dislocations, sprains, and strains	29	13.9
Burns and corrosion	27	12.9
Cuts	23	11.0
Fractures	14	6.7
Concussion and internal injuries	5	2.4
Amputation	2	0.9
Acute poisoning or infections	1	0.5

### Data analysis

The collected data were cleaned, coded, entered, and analyzed using Statistical Package for Social Sciences (SPSS). Continuous variables were described by mean and standard deviation (SD), and categorical variables were described by proportion (%). Chi‑squared tests were used for comparisons of categorical variables. The significance level was set to less or equal to 0.05. Binary and multivariate logistic regression analyses were performed to assess the potential determinants of occupational injuries while controlling for potential confounders.

### Ethics

Ethical approval was obtained from the Muhimbili University of Health and Allied Science institutional review board on 31/03/2022 with approval number MUHAS‑REC‑03‑2022‑1061. Permission letters for all managers of the selected factories were distributed to the workers. All participants were informed about the study and gave their written consent before the interview was performed, and confidentiality regarding their information was underlined. The written information and consent forms were in Swahili, the local language. The study has been carried out in accordance with the Declaration of Helsinki.

## Results

### Description of study participants

Of 381 workers, 321 participated in the study. All were men. The mean age of respondents was 33 years. The married participants comprised 74.1%, and about 76.3% had less or equal to 4 working years. Many of the iron‑ and steelworkers (76%) worked for more than 10 hours per day (Table 1). All workers had a break of 1 hour during each working day.

### Prevalence of occupational injuries

A total of 221 (68.8%) of study participants reported having experienced at least one injury at work in the 12 months preceding the survey. Of these, 65.1% had had injuries that restricted them from working at least 1 day (Table 2), and these were defined as occupational injuries. A large part of the injuries were superficial (abrasions, blisters, contusions, puncture wounds) (110 [52.6%]), followed by dislocation, sprain, and strain injuries, which had been experienced by 13.9%. Burns and corrosion had been experienced by 12.9% and cuts by 11%. Also, fractures had been experienced, and two persons had even had amputations (loss of fingers) (Table 2). The most common location of injury was on the upper extremities (Table 2). A total of 89 (42.6%) of the injured respondents faced an occupational injury once; out of injured respondents, 135 (64.6%) reported being hospitalized or lying in bed at home due to the injury, and the majority (83 [77.6%]), had been hospitalized for 1–6 days. Most workers (192 [91.9%]) had their occupational accident in their usual work area, and most of the injuries took place in the afternoon (121 [57.9%]).

### Factors associated with occupational injuries

When comparing injured and non‑injured workers using chi‑squared tests, there was a significant difference between the groups regarding working years, working hours, work section, and shiftwork (Table 3). There was no difference between the groups regarding age, education level, marital status, or industry site (Table 3).

**Table 3:** Comparing workers from iron and steel industries in Tanzania, with and without occupational injuries the past year (*n* = 321).

VARIABLE	WORKERS WITH OCCUPATIONAL INJURY IN THE PAST YEAR (*N* = 209)	*X*^2^*	*P*‑VALUE
**Age group (years)**	*n (%)*		
18–30	117 (65.4)	0.029	0.986
31–44	69 (64.4)		
≥ 45	23 (65.7)		
***Education***			
Never been in school	12 (63.2)	2.105	0.967
Primary school	86 (64.7)		
Secondary school and above	111 (65.7)		
***Marital status***			
Never married	55 (65.5)	2.976	0.797
Married	154 (64.7)		
***Working years***			
1–4 years	169 (69)	6.824	0.009
> 4 years	40 (52.3)		
***Working hours per day***			
≤ 10 hours	38 (49.4)	11.073	0.001
> 10 hours	171 (70.1)		
***Section***			
Furnace	81 (58.3)	5.043	0.025
Rolling mills	128 (70.3)		
***Shiftwork***			
Yes	129 (70.1)	4.744	0.029
No	80 (58.4)		
***Industry site***			
A	73 (73.7)	5.047	0.168
B	35 (58.3)		
C	58 (63)		
D	43 (61.4)		

*X^2^ = Chi‑squared test.

Variables from the chi‑squared comparisons (Table 3) with a *p*‑value of less than 0.25 were included in a multivariate logistic regression analysis. Here, the dependent variable was having experienced an occupational injury the past year, and the independent variables were working years, working hours, work section, and shiftwork. The results from this analysis show that working for more than 10 hours (adjusted odds ratio [AOR] = 2.54, 95% CI [1.46–4.41]) was the only significant predictor of occupational injuries in the iron and steel industries (Table 4). According to our data, 244 workers reported working for more than 10 hours. Out of these, 112 had experienced an occupational injury during the afternoon.

**Table 4:** Multivariable logistic regression analysis of factors associated with occupational injuries.

VARIABLES	CRUDE ODDS RATIO (95% CONFIDENCE INTERVAL)	ADJUSTED ODDS RATIO (95% CONFIDENCE INTERVAL)
***Working years***		
≤ 4 years	0.50 (0.29–0.85)	0.55 (0.29–1.03)
> 4 years	1	1
***Working hours per day***		
≤ 10 hours	1	1
> 10 hours	2.40 (1.42–4.06)	2.54 (1.46–4.41)
***Sections***		
Furnace	1	1
Rolling mills	1.70 (1.07–2.70)	1.59 (0.94–2.67)
***Shift work***		
Yes	0.6 (0.38–0.95)	0.53 (0.26–1.10)
No	1	1
***Industry***		
A	1	1
B	0.57 (0.29–1.09)	0.79 (0.37–1.74)
C	1.14 (0.56–2.29)	1.15 (0.49–2.68)
D	0.93 (0.49–1.77)	0.70 (0.34–1.43)

## Discussion

The 12‑month prevalence of occupational injuries in this study was 65.1%. This is similar to the findings in a similar cross‑sectional study in the iron and steel industries from Iran, which reported an injury prevalence of 61.7% [10]. However, the figures from both the Iranian and the present study show a higher prevalence of occupational injuries than reported in the iron and steel industries of a few other countries, for instance, Ethiopia (38.4%) [22], India (28.12%) [15], and Turkey (28.40%) [23]. These three studies were also cross‑sectional studies and gathered information through interviews, similar to the present study. The reason for the lower prevalence reported in these other studies is unclear. It could be due to different working conditions, which were not well described in these previous studies. Additionally, variations in national safety culture and occupational health and safety (OHS) practices might also play a significant role.

In the present study we found that workers who are working for more than 10 hours per day had a higher risk of getting occupational injuries. This finding is in line with several studies, as shown in a review of shift work and extended working hours [24], as well as in an American study that showed a relationship between long working hours and a high risk of occupational injuries among American metalworkers [25]. The higher risk of occupational injuries due to extending working hours can be attributed to exhaustion and stress, which affect the concentration of the workers and make them more prone to accidents. However, the number of injuries among those working more than 10 hours per day was similar to that of the daytime and afternoon.

The most injured body parts in the present study were the upper extremities (42.1%). This finding is consistent with several other studies, for instance, studies from Brazil [16, 26, 27], Iran [28], Ethiopia [2, 13], and India [11, 29]. This can be explained by the fact that the hands and arms are more involved in manual work in the metal industry than other parts of the body, and hence have a higher possibility of exposure to hazardous substances and dangerous activities when the worker is not fully protected.

Most of the occupational accidents in the present study occurred at their usual working site, and very few happened during work‑related travel. These findings are in line with data from the Australian Bureau of Statistics, which showed that, in 2021–2022, the most work‑related injuries occurred at the regular workplace [30]. Also, in the Tanzanian metal industry, the workers do not travel much outside work premises.

Most of the injuries in the present study occurred in the afternoon, with fewer injuries in the morning. This contrasts with studies from Ethiopia [2] and Iran [28], which found that injuries were more common in the morning. This difference might be explained by the geographical location. The selected industries in Tanzania are in a tropical coastal climate, which experiences very high temperatures during the day. This increases the rate of fatigue and exhaustion in the afternoon, thereby influencing the rate of injury occurrence.

Workers with less work experience were more likely to sustain occupational injuries than those with more years of experience. This finding is in line with a study done in Brazil [16], and may be due to a lack of job training and health and safety information among newer workers. Also, more experienced workers may have a higher awareness of workplace risk factors.

The present study was performed in Tanzania, and it is likely that most findings are relevant also to other similar factories in the metal and steel industries in the country. Additionally, the findings might be applicable to the metal and steel industries in other countries with similar manufacturing processes. However, one should be careful when generalizing the findings to the other manufacturing industries owing to the variation in setting and manufacturing processes. Nonetheless, similar findings might be observed in comparable factory sites in other low‑income settings on the African continent.

## Limitations and Strengths of this Study

The study has some limitations, as it was designed as a cross‑sectional study. A longitudinal study would have given more information but would also have demanded more resources to be performed. This weakness of the study was reduced by asking for information about the preceding year to have a lengthy information period. However, this introduced the risk of information bias, as the workers might not remember or might incorrectly remember the accidents and injuries that had happened. This might have led to both under‑ and over‑reporting; this is difficult to know. Another limitation is that the workers might be concerned about providing information to the researchers regarding their injuries and the workplace conditions, fearing that their information may not be kept confidential. This limitation was minimized by using private rooms for the interview. Despite this limitation, the study has yielded valuable information for accident prevention. The response rate was high, as the workers were very willing to provide information. Only one of the four factories had a lower response rate. We unfortunately have no information about why this was the case.

The ILO‑validated tool was used to collect information about self‑reported injury prevalence and personal and risk factors. Asking the workers was the only possible method of obtaining information about occupational injuries, as Tanzania has no official statistics on this topic, and the industries have no systematic reporting system.

The study demonstrates a relationship between long working hours (> 10 hours daily) and occupational injuries. This is a factor that needs to be considered in these industries, and long working hours should be avoided. The number of workers might have been too low to detect other significant relationships, and the lack of association with other factors might need to be interpreted with caution. However, the high prevalence of occupational injuries suggests a need to improve safety and health in these working environments. Improvements in this industry can be made through many different actions. Technically, for instance, the use of induction furnaces might be discussed. Different types of furnaces might eliminate the need for sorting, and the handling of the metal scrap as well as the temperatures might be reduced. Another option for improvement at this type of workplace is to avoid work during afternoons and at night. Also, other factors might be considered to reduce the injury rate in this type of working environment, such as more breaks or temperature reductions [31]. Further studies on occupational injuries are needed to learn more about these dangerous environments and how they can be improved.

## Conclusion

The prevalence of occupational injuries in the iron and steel industries in Tanzania was 65.1%. Working for more than 10 hours per day is a significant predictor of occupational injuries.

## Data Availability

Data are not available to anyone other than the authors due to restrictions by the ethical boards.
